# Motivations underpinning honeybee management practices: A Q methodology study with UK beekeepers

**DOI:** 10.1007/s13280-022-01736-w

**Published:** 2022-05-19

**Authors:** Fay Kahane, Juliet Osborne, Sarah Crowley, Rosalind Shaw

**Affiliations:** Environment and Sustainability Institute, University of Exeter, Penryn Campus, Penryn, TR10 9FE Cornwall UK

**Keywords:** *Apis mellifera mellifera*, Co-production, Natural beekeeping, Sustainable beekeeping, *Varroa*, Wild pollinators

## Abstract

Beekeepers are central to pollinator health. For policymakers and beekeeping organisations to develop widely accepted strategies to sustain honeybee populations alongside wild pollinators, a structured understanding of beekeeper motivations is essential. UK beekeepers are increasing in number, with diverse management styles despite calls for coordinated practice to manage honeybee health. Our Q methodology study in Cornwall, UK, indicated five beekeeping perspectives; conventional hobbyists, natural beekeepers, black bee farmers, new-conventional hobbyists and pragmatic bee farmers. Motivations can be shared across perspectives but trade-offs (notably between economic, social responsibility and ideological motivations) result in differing practices, some of which counter ‘official’ UK advice and may have implications for pollinator health and competition. Honeybee conservation emerged as a key motivator behind non-conventional practices, but wild pollinator conservation was not *prioritised* by most beekeepers in practice. Q methodology has the potential to facilitate non-hierarchical collaboration and conceptualisation of sustainable beekeeping, moving towards co-production of knowledge to influence policy.

**Supplementary Information:**

The online version contains supplementary material available at 10.1007/s13280-022-01736-w.

## Introduction

The Western honeybee *Apis mellifera* is a critical insect pollinator in natural habitats (Hung et al. 2018) and for crops (Aizen and Harder 2009; Breeze et al. 2011). Changes in honeybee colony density in Europe parallel beekeeper density (Potts et al. 2010), and colony numbers often correlate with political and socioeconomic systems (Moritz and Erler 2016). Although honeybees face intense pressures (Goulson et al. 2015), colony losses can be mitigated by beekeepers and recorded honeybee declines are regional but not global (Aizen and Harder 2009). In England and Wales, the number of registered beekeepers has risen from c.16 000 in 2010 to c.42 000 in 2020 (DEFRA & Welsh Government 2020), with beekeepers crucial for the maintenance of UK honeybee populations as colonies are unlikely to survive long term outside managed hives (Thompson et al. 2014).

*Apis mellifera* is one of over 20 000 bee species worldwide (IPBES 2016) and over 250 bee species in the UK (Falk and Lewington 2015). There is international scientific consensus that wild pollinators are in decline (IPBES 2016), and growing evidence that large-scale beekeeping operations can result in disease transmission and forage competition between honeybees and wild pollinators (Lindström et al. 2016; Mallinger et al. 2017; Bartlett et al. 2019). This has led to calls for reduction of managed honeybee colony numbers on farms (Garibaldi et al. 2014) and management of honeybee stocking densities in protected areas (Henry and Rodet 2018, 2020). Beekeepers are therefore critical not only for maintenance of UK honeybee populations, but also in ensuring honeybee management practices do not adversely impact wild pollinators.

The *Varroa destructor* mite is thought to have decimated UK honeybee colonies since its introduction in 1992 (Martin et al. 2012), with little evidence of any wild (unmanaged) honeybee colonies left in the UK (Thompson et al. 2014). *Varroa* increases honeybee mortality through reduced fitness and transmission of viruses among honeybees and wild pollinators (Fürst et al. 2014; Manley et al. 2015; Bailes et al. 2018). The extent of virus transmission is not yet fully understood, but *Varroa* has severe economic implications for individual beekeepers (Breeze et al. 2017) and industry (Cook et al. 2007), and likely negative implications for wild pollinators (Manley et al. 2015).

### ‘Official’ UK Honeybee management advice

The National Bee Unit (NBU) is an advisory, research and regulatory body delivering the English and Welsh Government’s ‘Healthy Bees Plan 2030’ (DEFRA & Welsh Government 2020). The British Beekeeping Association (BBKA) represents around 25 000 beekeepers via local organisations, delivering education and funding research. NBU and BBKA advice of relevance to this study stipulates that all UK beekeepers should inspect their colonies weekly for signs of disease, monitor and treat for *Varroa* and diseases using approved chemical treatments and/or hive manipulations to keep below the ‘economic injury level’, and minimise loss of swarms through approved hive manipulations (more detailed advice available at www.nationalbeeunit.com). Wild pollinators are mentioned several times in the Healthy Bees Plan (DEFRA & Welsh Government 2020), mainly regarding disease transmission risks, but at present, there are no standard guidelines for managing honeybees to coexist sustainably with wild pollinators in the UK.

### Different approaches to *Varroa* management

*Varroa* can be managed using different chemical treatments and hive manipulations, with beekeepers in the US favouring different management techniques based upon their concept of ‘stewardship’, resulting in ‘treatment sceptics’ and ‘treatment adherents’ (Thoms et al. 2018). Management practices have been correlated with US beekeeper philosophy (conventional, organic or natural) and size of operation (Underwood et al. 2019). Andrews (2019) describes ‘natural beekeepers’ in the US who do not treat for *Varroa*, and ‘not-so-natural’ beekeepers, who seek to improve the genetic fitness of honeybees through selective breeding. Research supporting these approaches maintains that *Varroa* treatment reduces opportunity for honeybees to evolve adaptive strategies through natural selection, thus reducing their genetic fitness over the long-term (Conte et al. 2007; Locke and Fries 2011).

### Knowledge co-production for sustainable beekeeping

Sustainability in beekeeping is a complex concept underwritten by social, ecological and economic opportunities and constraints. Inconsistencies in the literature and strong, polar opinions on the ground have created a diversity of opinion and practice, and misunderstanding within the beekeeping community and among policymakers can be high (Phillips 2014; Andrews 2019), with frustrations around ‘knowledge hierarchies’ whereby the opinions of researchers and some beekeepers are prioritised above others (Maderson and Wynne-Jones 2016). A comprehensive review (Guichard et al. 2020) revealed no scientific evidence that either natural or artificial honeybee selection approaches have long-term success in improving *Varroa* resistance/tolerance. However, beekeepers and academics are questioning the prioritisation of reductionist science (reduction of complex phenomena to simple constituents) over holistic beekeeper knowledges in diagnosing bee health issues (Kleinman and Suryanarayanan 2013), and advocating co-production of knowledge whereby beekeepers are equal partners in the design and implementation of academic research (Suryanarayanan et al. 2018; Kleinman and Suryanarayanan 2020).

## Theoretical framework

Q methodology was selected as suitable for a grounded-theory, participant-led approach to this research, whereby themes and concepts emerge through inductive analysis of collected data. The method systematically investigates subjective viewpoints on a given issue or ‘discourse’ and utilises inverted factor analysis alongside qualitative interpretation to identify clusters of shared opinion which can be described holistically (Brown 1980; Watts and Stenner 2012; McKeown and Thomas 2013). Q method is increasingly used in conservation research (Zabala et al. 2018) to assist the development of policies that are acceptable to stakeholders (Crowley et al. 2020), allow critical reflection by researchers and practitioners (Sandbrook et al. 2013) and diplomatically address conflict on contentious issues (Newth et al. 2019). This study, funded by the Halpin Trust, has been undertaken across the ecological and social sciences and is the first globally to apply Q methodology to an issue that has previously predominantly been explored through an ecological lens.

### This study aims the following:

1. Use Q methodology to generate a structured understanding of motivations underpinning beekeeping management practices in our case study area of Cornwall, southwest UK.
2. Investigate motivations underpinning management practices that contradict official NBU advice and/or may adversely impact wild pollinator populations.
3. Explore the potential of Q methodology as a means of co-production of knowledge around sustainable beekeeping.

## Materials and methods

Q method consists of three stages: co-creating a ‘Q set’ of statements relating to beekeeping motivations, undertaking a ‘Q sort’ whereby participants rank statements on a scale from strongly agree to strongly disagree, and finally, performing statistical and qualitative analyses on the data.

### Co-creating the Q set

Semi-structured interviews covering motivations for keeping bees, management priorities and goals, perceived threats, and concepts of ‘sustainable beekeeping’ were undertaken with eleven beekeepers in June 2019 (Appendix S1). Interview participants were purposively selected to represent a range of age, gender, experience, geographic location (within Cornwall) and beekeeping management preferences. This was achieved through contacting individuals associated with different beekeeping groups (see Table 1) and subsequent snowball sampling. Selection was also undertaken through participant observation at two major beekeeping events in Cornwall (the Royal Cornwall Show and annual ‘Bee Health Day’). Ten interviews were recorded with permission using the ‘Voice Recorder’ app (Tapmedia Ltd., 2015; Version 3.8), transcribed by hand and analysed for common themes, leading to an inductive categorisation of six motivational themes in beekeeping: ‘personal’, ‘economic’, ‘influences & communication’, ‘social responsibility’, ‘bee welfare’ and ‘ideology’. A structured Q set of statements was developed from these data, along with selected beekeeping blogs and magazines, to ensure balanced coverage of the full diversity of motivations (Watts and Stenner 2012). Each Q set statement formed the second half of the opener ‘As a beekeeper, I do what I do because…’ (for example, ‘…I enjoy it’). The initial set of statements were reviewed with one beekeeper (who was also interviewed for the Q sort) and two ecologists (who were not interviewed for the Q sort). Minor revisions were made to several statements to improve clarity, and several were removed to avoid repetition, resulting in a final Q set of 43 statements (Table 2).

**Table 1:** UK beekeeping groups and associations, used as a basis for purposive selection of participants

Beekeeping group	Description	Website
British Beekeeping Association (BBKA)	Representing around 25 000 beekeepers via local organisations; promoting ‘official’ management practices as advised by the National Bee Unit (NBU)	www.bbka.org.uk
Bee Farmers Association	Representing over 500 bee farmers in the UK, who each have over 40 colonies (up to thousands) and produce honey in bulk for sale	www.beefarmers.co.uk
Natural Beekeeping Trust	Promoting a variety of ‘bee-centred’ approaches that reject many BBKA and NBU-advised practices	www.naturalbeekeepingtrust.org
Bee Improvement and Bee Breeders Association (BIBBA)	Aiming to conserve the European dark honeybee or ‘black bee’ *Apis mellifera mellifera* (*Amm*), often through genetic research and selection techniques	www.bibba.com

**Table 2:**
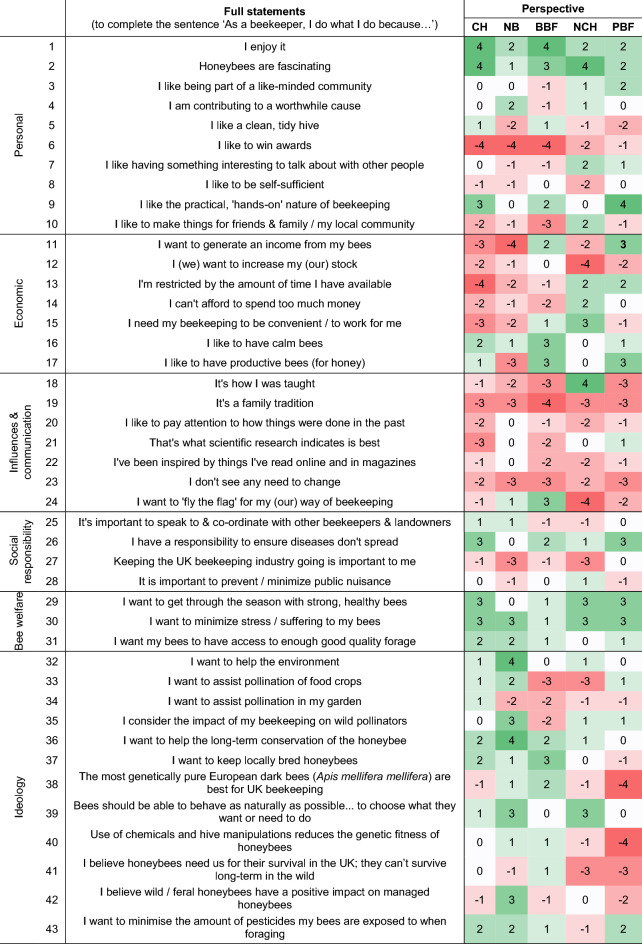
Full Q set statements grouped by motivational theme (first column), with representative Q sort rankings for each of the five perspectives. +4 (green): strongly agree. -4 (red): strongly disagree

*CH* conventional hobbyists, *NB* natural beekeepers, *BBF* black bee farmers, *NCH* new-conventional beekeepers, *PBF* pragmatic bee farmers

### Undertaking the Q sort

**Fig. 1:**
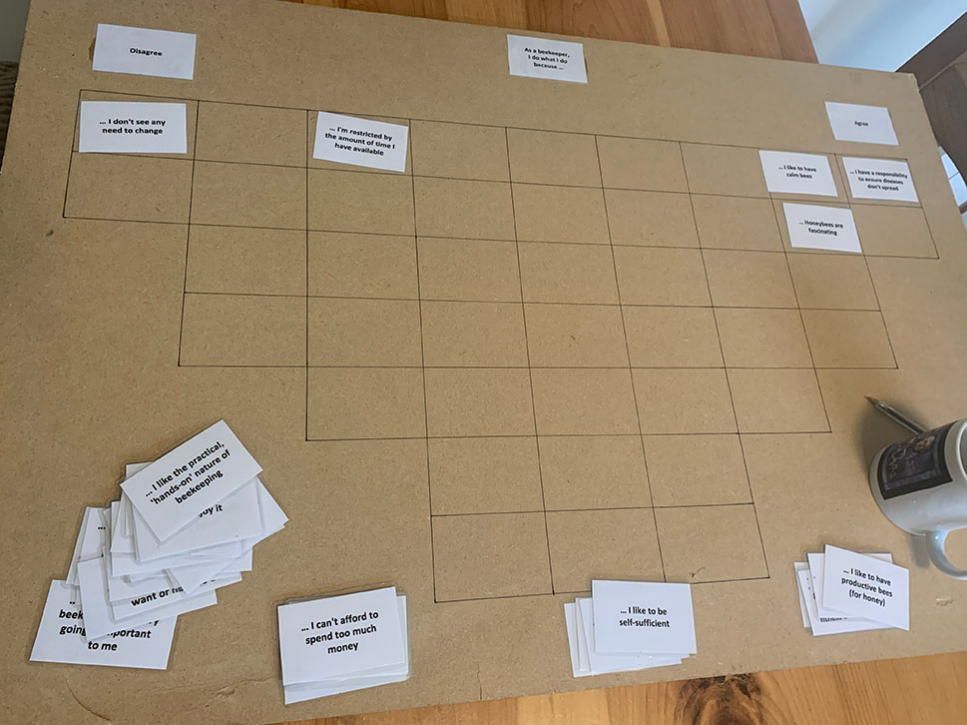
Photograph of a Q sort in progress illustrating the grid layout for placement of the 43 Q set statement cards, ranked horizontally from -4 (strongly disagree, far left column) to +4 (strongly agree, far right column). Photo credit: F Kahane

Each statement was printed onto credit-card-sized laminated cards, and a grid prepared to allow relative ranking of statements (Fig. 1) for the main Q-sort interview process. Twenty-one purposively selected participants (including eight from the initial interviews) were interviewed from July to September 2019 through snowball sampling from the initial interviewees, to represent a range of age (20 to over 70), years’ experience (0.5 to over 70), number of colonies (1 to over 130), gender (10 females, 11 males), location (across Cornwall from Penwith to the Devon border) and association with different beekeeping groups (Table 1). Participants were anonymised and gave informed consent following explanation of the purposes of the study (Appendix S2), with prior approval granted by the University of Exeter College of Life and Environmental Sciences (Penryn) Ethics committee. Participants sorted all cards into three initial piles of ‘agree’, ‘disagree’ and ‘neutral/need to think more’, before populating the array (grid squares), ranked horizontally from agree to disagree (no vertical order). During the Q sort, participants were encouraged to talk about their motivations in a recorded semi-structured interview, which included management questions covering hive inspections, *Varroa* treatment, source of stock and swarm management (Appendix S3).

### Analysis

**Fig. 2:**
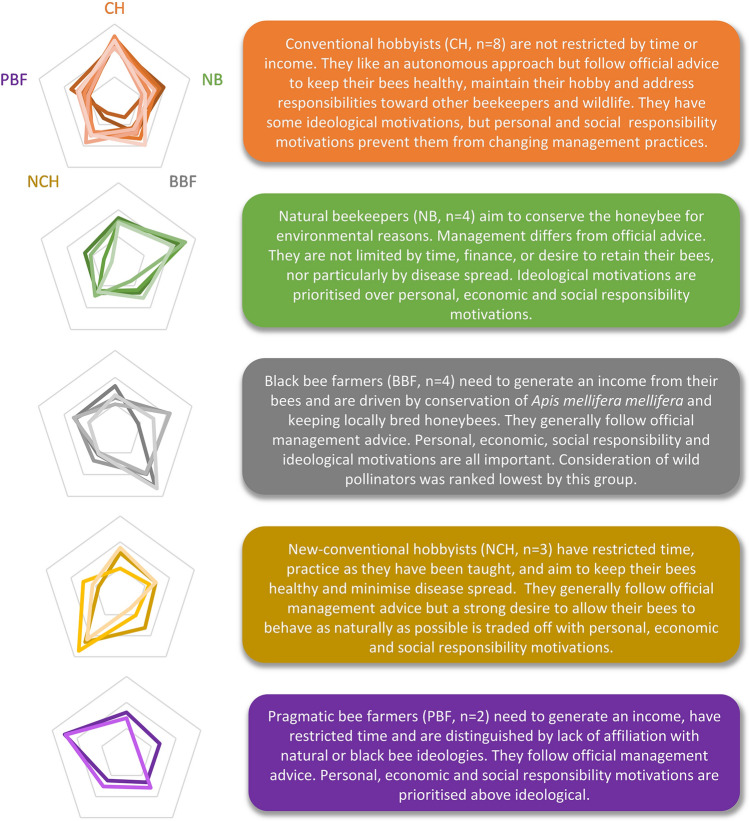
Radar plots illustrating the 'loading 'of each participant (coloured line, *n* = 21) on to each perspective (pentagon point, *n* = 5). The closer the coloured line is to the pentagon point, the more that participant agrees with that perspective

The twenty-one Q sorts were tabulated into a matrix of Q sort (21 columns) by Q set motivation statement (43 rows), with each cell populated by a ranking from -4 (most strongly disagree) to + 4 (most strongly agree). These data were intercorrelated and subjected to an inverted (by-person/Q sort) factor analysis using the qmethod package (Zabala 2014) in RStudio (Version 1.2.1335) (output in Appendix S4). Extraction of up to seven factors (considered to be a workable maximum in Q methodology; Watts and Stenner 2012) were investigated for fit using both qualitative and quantitative criteria, such that the extracted factors must have eigenvalues > 1 (to satisfy the *Kaiser–Guttman* criterion; see Watts and Stenner 2012), two or more significantly loading Q sorts at the 0.1 level (Humphrey’s rule; Brown 1980) and a clear qualitative explanation. Combined with a scree plot of eigenvalues which had a slight elbow at 5, these criteria led to five factors being extracted and subjected to Varimax rotation and Principle Components Analysis. Five representative Q sorts were generated through automatic flagging of significantly loading participants onto each factor and calculation of weighted means (z-scores) to show relative position of each statement within a factor (Table 2). z-scores were also used to identify distinguishing statements (where z-scores differ significantly between factors at the 0.05 level) and consensus statements (no significant difference in z-scores between any pair of factors at the 0.05 level) (Zabala, 2014). A factor loading was calculated for each participant in relation to all five factors, showing how close they are to each representative Q sort, and presented as radar plots (Fig. 2).

Twenty recorded interviews (ranging from half an hour to over two hours) were transcribed (one non-recorded interview was transcribed from hand-written notes) and entered into NVivo 12 Plus. Transcriptions were coded according to Q set statements and the six motivation themes, along with other emerging themes, in an abductive approach to thematic analysis (Watts and Stenner 2012; Chapter 2). A description of five ‘typical’ beekeeping perspectives was generated through a ‘structured approach’ to analysis, whereby a written perspective is methodically created for each factor based upon detailed examination of Q-sort statement ranking (Watts and Stenner 2012; Chapter 7), then enhanced and contextualised using thematic analysis of qualitative interview data.

## Results

Five factors (henceforth ‘perspectives’) were extracted, explaining 71% of the variance. The full Q set of statements plus representative Q sort for each perspective is shown in Table 2, grouped by six motivational themes (personal, economic, influences & communication, social responsibility, bee welfare and ideology). There were nineteen distinguishing statements; notably the majority were found within the motivation themes of economic (six) and ideology (eight). Most notable of the three consensus statements was that all groups felt open to changing management practices. Figure 2 gives a visual representation of how closely each individual participant corresponds to each of the five perspectives, along with a summary description of each perspective.

**Fig. 3:**
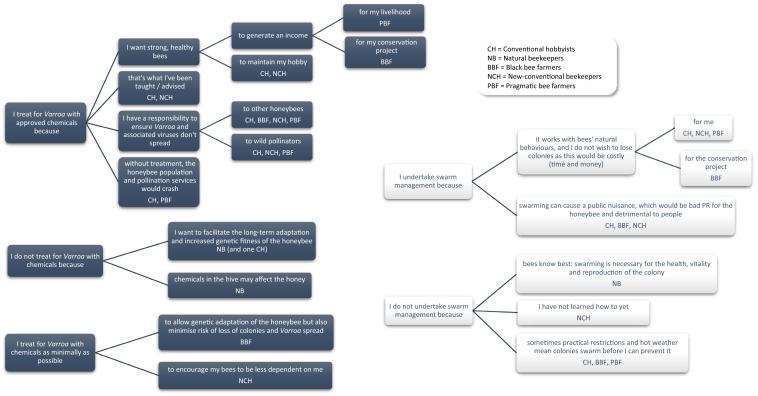
Flow charts illustrating motivations underpinning *Varroa* management decisions (blue boxes) and swarm management (white boxes) based on qualitative interview data

Despite differences in motivations between the groups, there is also overlap and fluidity; motivations can be shared across groups but conflicting demands result in trade-offs when it comes to management decisions. Table 3 summarises relative prioritisation of motivational themes for each perspective, detailing conflicting motivations that each individual beekeeper must trade off (through prioritisation of one motivation at the expense of another), resulting in different management practices (in this example, *Varroa* treatment). Figure 3 further illustrates these trade-offs, providing summary flowcharts of qualitative data around motivations underpinning the two key management practices of *Varroa* treatment and swarm management. In the following descriptions of each perspective, Q set statement number and ranking are given as appropriate, for example (18: + 4) refers to statement 18 being ranked as most strongly agree (+ 4; see Table 2). Distinguishing statements (where z-scores differ significantly at the 0.05 level between factors) are in bold (e.g. **18: + 4**). A summary of management practices as elicited from the semi-structured interviews is provided in Appendix S5.

**Table 3:** Trade-offs taken by each perspective under conflicting motivations. Conservation ideology can encourage non-conventional practices (eg. no Varroa treatment). Relative rank of two conflicting statements is highlighted, alongside management decision

	Motivational themes leading to conventional management	Motivational themes leading to non-conventional management	Ranking of motivation to minimise disease spread (statement 26)	Ranking of motivation to conserve the honeybee (statement 36)	Management decision: treat for *Varroa*?
Conventional hobbyists	Personal, social responsibility	Ideological	+ 3	+ 2	Yes
Natural beekeepers		Ideological	0	+ 4	No
Black bee farmers	Personal, economic, social responsibility	Ideological	+ 2	+ 2	Yes (minimal)
New-conventional hobbyists	Personal, economic, social responsibility	Ideological	+ 1	+ 1	Yes (minimal)
Pragmatic bee farmers	Personal, economic, social responsibility		+ 3	0	Yes

### Five beekeeping perspectives

**Conventional hobbyists** (n = 8, eigenvalue 4.5, explained variance 22%) are not restricted by time or money (**13: − 4**, 14: **− **2) and are fairly unified in following conventional management techniques. They like an autonomous approach but follow NBU advice to keep their bees healthy (29: + 3) and maintain enjoyment of their hobby (2: + 4, 1: + 4). There was some agreement with motivations behind natural beekeeping approaches (39: + 1, 40: 0), but their preferences for a hands-on approach (9: + 3), wanting to minimise disease spread (26: + 3) and ideally get a honey return (**17: + 1**) prevent them from changing management practices to pursue natural beekeeping ideologies. They feel bees should be able to behave as naturally as possible (39: + 1) but beekeepers need to ‘nudge’ them as necessary. Ultimately, this group (except one self-described natural beekeeper) treat for *Varroa* prophylactically because other motivations are stronger for them:

I wish I didn’t (use *Varroacides*), I wish I was braver (but) … I don’t want to let my bees die because they’re good bees.

Although motivated by long-term conservation of the honeybee (36: + 2), this does not rank as highly as colony health (29: + 3). Minimising stress and suffering is important (30: + 3), resulting in a desire to carry out inspections as carefully as possible. Although generally considering that wild/feral honeybees have a negative impact on managed honeybees (42: − 1) due to *Varroa*/disease spread, it was sometimes acknowledged that feral bees may provide fitter drones. Some believe wild/feral colonies die within 3 years and others believe such colonies survive for many years (41: 0). They are keen to keep locally bred honeybees (37: + 2), mainly to reduce risk of disease from imports, but are not motivated by keeping the subspecies *Apis mellifera mellifera* (henceforth *Amm*) (38: − 1). Although aware of the potential impact of their beekeeping on wild pollinators in terms of disease spread and forage competition, consideration of wild pollinators is not generally a motivator for management practice (35: 0). With a small number of notable exceptions, this group disagree that scientific research influences how they practice (21: − 3). They learn from their bees and other beekeepers and, in common with all other groups, feel that beekeepers should be open to change (23: − 2).

**Natural beekeepers** (*n* = 4, eigenvalue 3.6, explained variance 17%) are motivated by conservation ideologies; helping the environment and long-term conservation of the honeybee (**32: + 4, 36: + 4**) through facilitation of natural selection via exposure to stressors. Significantly, long-term conservation of the honeybee (**36: + 4**) is ranked markedly higher than health of current colonies (29: 0). Willing to lose colonies to swarming and/or *Varroa*, they do not want or need to generate an income or honey return (11: − 4, **17: **− **3**). They also prefer a more hands-off approach (9: 0) and despite agreeing that honeybees are fascinating (1: + 2; 2: + 1), these motivations are ranked lower than in other groups, indicating they are driven by their perception of the bees’ needs, not their own. Deeming regular internal inspections a cause of stress and suffering (30: + 3), they believe bees should be able to behave naturally (39: + 3) and that use of chemicals and hive manipulations reduces genetic fitness (40: + 1):

I strongly believe that any sort of chemical or hive manipulation is just interference with natural processes and reduces the nature of the colony to adapt – it’s like a sticking plaster on a wound.

Ranking of responsibility to ensure diseases do not spread is the lowest of all groups (**26: 0**); keeping bees at lower densities is seen to mitigate the risk of disease spread. They strongly believe wild/feral honeybees have a positive impact on managed honeybees (**42: + 3**) and believe that honeybees are able to survive outside managed hives (41: -1). Keeping locally bred bees and *Amm* is supported (37: + 1; 38: + 1), but not through artificial selection. The overall ethos of natural beekeeping is promoted as a more sustainable balance with wild pollinators (35: + 3); allowing honeybees to swarm/die/find their own locations and densities. They are more interested in how things were done in the past and in scientific research (20: 0; 21: 0) than most other groups, are fairly keen to promote their way of beekeeping (**24: + 1**) and feel strongly about being open to change (23: − 3).

**Black bee farmers** (*n* = 4, eigenvalue 2.6, explained variance 12%) enjoy keeping bees (1: + 4, 2: + 3) and are both practically *and* ideologically focussed, with three of the four united by their desire to selectively breed the European dark honeybee or ‘black bee’ *Amm,* considered to be native to the UK (38: + 2, although one has more recently been questioning this aim). For all, keeping locally bred bees is important (37: + 3) and they like to experiment hands-on with the bees’ capabilities (9: + 2). Having calm (**16: + 3**) and productive bees for honey (17: + 3) is also important, along with generating an income (11: + 2), but not at the expense of colony health (29: + 1) or honeybee conservation (36: + 2):

To me, keeping the native bee is maintaining longevity of that DNA strand that nature had created for the purposes of the environment which it found itself in … if I’m here for one thing it’s to pass on those genes … and for (humans) to benefit.

In common with the natural beekeepers, they agree that chemicals and hive manipulations reduce genetic fitness (40: + 1), but feel responsible regarding disease spread (26: + 2) so they treat for *Varroa* prophylactically on productive colonies but only as necessary on breeder colonies:

Part of me thinks (not treating for *Varroa*) is a brilliant idea, part of me knows I’ve lost 40% before and it’s too high a risk.

This group do not tend to consider wild pollinators (**35: − 2**) in their beekeeping, due to their focus on conservation of *Amm*. They seek to promote their way of beekeeping (**24: + 3**) and prefer to be taught by bees through experience rather than scientific research (21: **− **2). In common with other groups, they feel it is important to be open to change (23: **− **3).

**New-conventional hobbyists** (*n* = 3, eigenvalue 2.4, explained variance 11%) find honeybees fascinating (2: + 4), enjoy the personal benefits of hobby beekeeping (3: + 1, 7: + 2, **10: + 2**) and are the only group to agree that they practice as they have been taught (**18: + 4**). They generally follow official NBU management advice, need their beekeeping to be convenient (15: + 3) and can find practical requirements difficult (9: 0) as they have restricted time (13: + 2) and available finance (14: + 2). They aim to keep their bees alive, healthy and under minimal stress (29: + 3, 30: + 3), consider implications of disease spread (26: + 1) but trade this off with a strong desire to allow their bees to behave as naturally as possible (39: + 3); in contrast with conventional hobbyists who rank responsibility to minimise disease spread higher than allowing bees to behave naturally. They seek to minimise *Varroa* treatments, but their main drive is to keep their bees alive and healthy and not cause a nuisance (28: + 1) to others. New-conventional hobbyists strongly disagree that honeybees need humans for survival (41: − 3) and although agreeing in principle that chemicals and hive manipulations may reduce genetic fitness and wild/feral honeybees have a positive impact upon managed bees, this did not influence practice (42: -1; 40: -1). Keeping locally bred bees and superiority of *Amm* were not rated highly (37: 0, 38: -1); not because of outright disagreement but because it does not influence their beekeeping. They have no desire to promote their way of beekeeping (24: -4); mainly due to some self-doubt, concerns around possible negative impacts of beekeeping on wild pollinators (35: + 1) and awareness of the complexity of ideological issues, and again feel it is important to be open to change (23: **− **2):

Right now, I want to look after (my bees), but the scientific grand scheme would be to (get weak ones) out of the gene pool. I feel like I contradict myself all the time ... But if you look at things from different angles you’ll get different views.

**Pragmatic bee farmers** (*n* = 2, eigenvalue 1.9, explained variance 9%) greatly enjoy their hands-on trade (9: + 4) and need to generate an income (11: + 3). With restricted time (13: + 2), they practice conventional management techniques to maximise honey production (17: + 3) and minimise bee losses (29: + 3), bee stress (30: + 3) and disease spread (26: + 3). Of all the groups, they most enjoy being part of the beekeeping/bee farming community (3: + 2) and are most likely to be influenced by scientific research (21: + 1). Trade-offs are not as relevant for this group, as practical and economic considerations are prioritised above ideologies (32: 0, 36: 0, 39: 0). They believe honeybees are able to survive in the wild (41: − 3) but that wild/feral bees have a *negative* impact on managed bees (42: − 2), mainly due to *Varroa*/disease spread. Risk of reducing the genetic fitness of honeybees does not influence their beekeeping (**40: **− **4**), but one participant did have sympathy with the idea of allowing natural selection:

In that one shouldn’t treat if it’s not necessary, that’s probably a good thing but … I need to think about cost-effectiveness.

They also disagree strongly that genetically pure *Amm* are best for UK beekeeping (**38: **− **4**), although for different reasons; one disagreeing in principle, valuing genetic diversity among local hybrids, while the other is supportive of such projects for the purposes of bee breeding, but needs a productive hybrid rather than a pure strain. Sourcing local stock is not prioritised (37: − 1). Aware of virus transmission and possible forage competition among managed honeybees and wild pollinators (35: + 1), they follow NBU advice. They have altered their practice from the way they were taught (18: − 3) and again, feel it is important to be open to change (23: − 3).

## Discussion

This study provides evidence of non-conventional beekeeping practices in the UK with motivations beyond productivity and health of current colonies, mirroring other studies globally (Andrews, 2019). Most notable in this context are motivations underpinning management practices that counter official UK (NBU and BBKA) advice to inspect hives weekly, treat for *Varroa* and diseases with approved chemicals and hive manipulations, and minimise loss of swarms (DEFRA & Welsh Government 2020).

The relative prioritisation of personal, economic, social responsibility and ideological motivations defines each perspective, but there is also overlap and fluidity; ideological motivations in particular can be shared across groups. This can lead to conflicting motivations held by individual beekeepers, requiring trade-offs when it comes to management decisions. For example, all beekeepers interviewed wished to minimise stress and suffering to honeybees, but for natural beekeepers this was through minimal inspection, whereas for conventional groups this was through regular inspection to check colony health:

In some ways, if I wanted to minimise stress and suffering I probably wouldn’t inspect them. But one of the main reasons I (inspect) every week is to … recognise if they’re hungry or suffering (Conventional hobbyist).

### Honeybee conservation drives lack of *Varroa* treatment and swarm management

The key motivation underpinning lack of *Varroa* treatment or swarm management is a belief that in-hive chemicals and manipulations reduce the genetic fitness of honeybees (Fig. 3), with implications for honeybee conservation. Natural beekeepers were the only group to rank this above the responsibility to minimise disease spread, and to this end they do not treat for *Varroa* (Appendix S5). Black bee farmers, who also agree with this statement but cannot afford to lose colonies, treat breeder colonies only if perceived *Varroa* load is particularly high, treating their productive colonies (those used to generate an income through honey) prophylactically. Breeder colonies are used to select and breed *Amm* queens, which are upheld as native to the UK (Carreck 2015), with claimed superior *Varroa* resistance/tolerance (Pinto et al. 2014; Ellis et al. 2018; Hassett et al. 2018). Interestingly, some participants within the conventional hobbyists, and all within the new-conventional hobbyists also tended to agree with the statement in interview, but it does not tend to influence *practice* due to the prioritisation of other motivations (responsibility to prevent disease spread, and desire for strong, healthy bees); providing depth and nuance to the documented ‘treatment adherent’–‘treatment sceptic’ divide (Thoms et al. 2018). The pragmatic bee farmers strongly disagree, with a clear view that they are livestock farmers and to that end need to prioritise current colony health and treat for *Varroa*. Imported honeybees are the main infection source of *Varroa-*transmitted viruses (Fürst et al. 2014) and all beekeeper groups in this study want to keep locally sourced honeybees *except* the pragmatic bee farmers, who prioritise productivity and calm temperament (although the pragmatic bee farmers in this study do in fact *currently* source local/UK stock; Appendix S5). Notably, there were also practical issues expressed around swarm management for some groups (Fig. 3); demonstrating that management is not always undertaken as planned, regardless of motivation.

Whether honeybees adapt to *Varroa* if left untreated, and indeed whether they can survive long term outside managed colonies in the UK, are both issues subject to scientific debate (Conte et al. 2007; Locke and Fries, 2011; Thompson et al. 2014; Loftus et al. 2016; Seeley, 2017). There were strong opinions on this among beekeepers; the black bee farmers believe that pressures of *Varroa* and disease, lack of forage or suitable cavities and intensive agricultural practices mean honeybees cannot survive long term outside managed colonies, which is a key motivation for breeding bees with better survival capabilities. The natural beekeepers believe honeybees can survive long term but are limited by the above pressures. New-conventional hobbyists and pragmatic bee farmers are confident that honeybees can and do survive long term outside managed colonies. The extent of *Varroa* transmission among managed and wild pollinators is not yet fully understood (Fürst et al. 2014; Manley et al. 2015) but economic implications, and potentially ecological implications, are severe (Cook et al. 2007; Manley et al. 2015; Breeze et al. 2017). Further research into the drivers and implications of different attitudes towards *Varroa* management is recommended, to co-create workable policies to sustainably manage the health of both managed and wild pollinator populations.

### Honeybee stocking densities and forage competition

Black bee farmers ranked the statement concerning impact of beekeeping upon wild pollinators lowest of all groups, reflecting their prioritisation of conservation of *Amm*. Reserves have been set up in Cornwall dedicated to encouraging the breeding of *Amm*, which are seen as overcrowded by some participants, with potential for forage competition between managed and wild bees (Lindström et al. 2016). There is broad agreement that keeping *locally bred* honeybees is preferable, due to disease risk from imported queens and stock (although pragmatic bee farmers prioritise productivity over local stock). However, concern was expressed that wider uptake of the *Amm* ‘message’ leads to more honeybee colonies located in areas dedicated for the protection of wildlife, which may be of detriment to wild pollinators of conservation interest.

The natural beekeepers believe that keeping honeybees at lower densities and allowing them to swarm/die out minimises competition with wild pollinators; a belief generally shared by the new-conventional hobbyists. The conventional hobbyists tended to feel that the scale at which they were operating was little cause for concern with regard to forage competition, and that practicing beekeeping had made them much more appreciative of the role of wild pollinators alongside managed honeybees. The pragmatic bee farmers did not see what else they could do for wild pollinators beyond managing swarming and treating for *Varroa*, and indeed there is no official UK advice on honeybee stocking densities. Exclusion of beekeeping from sensitive areas (Durant 2019) and calculation of maximum honeybee colony allowance in protected areas (Henry and Rodet 2018, 2020) have been put forward in Europe to minimise impacts upon wild pollinators, but implications of these suggestions for beekeepers remain unknown (Durant 2019). Access to good quality forage was seen as important by all perspectives but broadly outside beekeepers’ control due to the large foraging range of honeybees:

Forage (is) in the lap of nature and the farmer (Conventional hobbyist).

It is notable that, with the exception of natural beekeepers, consideration of wild pollinators did not emerge as a strong signal in this study, with beekeepers feeling that responsibility lies with land managers to provide more forage and nesting sites. With a grounded-theory, participant-led approach, ecologically sustainable beekeeping in terms of impacts upon wild pollinators was not at the forefront of beekeepers’ minds in this study.

### Working towards sustainable beekeeping: interdisciplinary research and co-production of knowledge

The Healthy Bees Plan 2030 (DEFRA & Welsh Government 2020) promotes ‘sound science and evidence’ along with ‘increased opportunities for knowledge exchange and partnership working’. Scientific research was predominantly viewed ambivalently or negatively by conventional hobbyists:

I can’t say I’ve read much scientific research … I keep bees because it’s a hobby and I’m not really interested in the science of it (Conventional hobbyist).

Black bee farmers also did not tend to rely on scientific evidence, preferring to utilise their own knowledge and experience, along with that of other trusted and respected beekeepers. Negative perceptions of knowledge hierarchies (Maderson and Wynne-Jones 2016) challenging beekeepers’ autonomy came into play, especially among conventional hobbyists:

(Scientists) come out with something (and say) ‘well this is what you should be doing’ … they try to tell you what you should do (Conventional hobbyist).

With beekeepers suspicious of motivations behind science-based beekeeping advice:

Sometimes science doesn’t give enough information as to how they arrived at that decision. (They) just expect me to believe it? Well no I don’t (Conventional hobbyist).

This contrasts with other studies whereby honeybee health stakeholders were found to look primarily to research articles for trusted information (Scott et al. 2013). Despite undertaking practices that go against NBU advice, natural beekeepers in this study were among the most supportive of research, with popular scientific influences (for example Seeley 2010) more prevalent than the spiritual influences described in other studies (Green and Ginn 2014). However, as a scientific approach has not yet provided evidence for large-scale solutions with regard to *Varroa* management (Guichard et al. 2020), there is a clear need for socially mediated co-production of knowledge (Jasanoff, 2004 as cited in Gustafsson et al. 2017). Collaboration of beekeepers with scientific researchers could facilitate coordinated, peer-reviewed reporting of beekeeping impacts upon both honeybees and wild pollinators, and generate reliable data to further environmental and economic goals. Further research into the potential impacts of beekeeping upon wild pollinators in the UK is needed; particularly natural beekeeping practices with regard to disease spread, and bee farming practices (stock density) with regard to competition for resources. It will be of paramount importance, however, that this is non-hierarchical to ensure collaborative framing of research questions at the outset, along with non-biased analyses and reporting. Calls have been made for transdisciplinary approaches to pollinator research (Bartomeus and Dicks 2019), and ecologists are increasingly seeking to influence policy around pollinator management (e.g. Dicks et al. 2016; Potts et al. 2016; Bartomeus and Dicks 2019; Kleijn et al. 2019), but a wider base from which to define research approaches is required. A truly interdisciplinary approach to understanding sustainability in beekeeping, co-produced with beekeepers holding a variety of perspectives, and designed and implemented in collaboration with trained social scientists, is fundamental to understand sustainability in beekeeping.

### Q methodology for co-production of knowledge

Frustration was evident in this study when motivations (especially ideology-driven) were only partially understood by others. Disagreement with the norm made some beekeepers feel alienated and less willing to engage with those who have a different opinion, resulting in tension within the community:

‘People say we’re a harbinger of infection’ (Natural beekeeper).

‘We are very defensive if anyone attacks us (and) says ‘your bees are killing off all the wild pollinators’ (Black bee farmer).

To this end, Q method was useful for self-reflection and relaxed consideration of motivations behind other ways to practice. Many participants commented on the method after interview, enjoying its hands-on nature and detailed examination of motivations both familiar and unfamiliar, although for one, the guided nature of card placement was perceived as restrictive. Several commented after interview that it had improved their understanding of alternative beekeeping practices, even those with which they still disagreed. The opportunity to engage face-to-face with a researcher to fully explain their perspective was uniformly welcomed by participants, who were happy to spend up to two hours in conversation. A strength of Q methodology is its lack of prioritisation of the dominant perspective; it is not the number of participants that have a particular perspective that is relevant, but a structured description of the *diversity* of perspectives. In this way, a broader conceptualisation of sustainability could be investigated through Q methodology. It can bridge qualitative–quantitative preferences among different researchers and allow beekeeper-led definitions of sustainability, along with wider exploration of potential solutions and barriers to those solutions. Co-production of knowledge in this way has been demonstrated to build the relationships needed to effect management and policy change, and increase uptake of recommendations (Lemos et al. 2018).

## Conclusions

This study demonstrates a diversity of motivations behind differing beekeeping practices in Cornwall, UK, organised into five different perspectives; conventional hobbyists, natural beekeepers, black bee farmers, new-conventional hobbyists and pragmatic bee farmers. Motivations can be shared across perspectives, but each beekeeper undertakes trade-offs between economic, social responsibility and ideological motivations which lead to differences in management practice. Natural beekeepers diverge most from official NBU advice regarding *Varroa* and swarm management, with implications for pollinator health, and although motivations underpinning natural beekeeping can be shared by other perspectives, economic and social responsibility motivations currently limit changes to beekeeping practice. Higher stocking densities of pragmatic and black bee farmers do not counter official advice but may impact wild pollinators through forage competition. Despite scientific evidence for negative impacts of beekeeping upon wild pollinators through disease spread and resource competition, consideration of wild pollinators did not emerge as a strong motivator in beekeeping practice. Further research into impacts of beekeeping on wild pollinators in the UK is needed, and it is recommended that this research is co-produced with beekeepers holding a diversity of perspectives. Q methodology was well received and has the potential to facilitate non-hierarchical conceptualisation of sustainability in beekeeping, collaboratively define research questions, methodological approaches and analytical frameworks, and build relationships needed to effect management and policy change, in a move towards co-production of knowledge around sustainable beekeeping.

## Supplementary Information

Below is the link to the electronic supplementary material.

Supplementary file1 (PDF 696 kb)

## Data Availability

Research data supporting this publication are available as supplementary information (Appendices S4 and S5). Due to ethical reasons, interview transcripts cannot be made openly available.
