# Supplementary material for: Motivations underpinning honeybee management practices: A Q methodology study with UK beekeepers
Source: Ambio. 2022 May 19;51(10):2155–68. doi: 10.1007/s13280-022-01736-w (PMC9378798; doi:10.1007/s13280-022-01736-w)
Supplement: Supplementary file 1 — Supplementary file1 (PDF 696 kb) [file 13280_2022_1736_MOESM1_ESM.pdf]

Ambio

Supplementary Information

This supplementary information has not been peer reviewed

Title: **Motivations underpinning honeybee management practices: a Q methodology study with UK beekeepers**

S1: Pre-pilot interview schedule and consent form (to determine the Q set)

S2: Participant Information Sheet and Consent Form

S3: Structured Management & Demographic Questions (forming part of the semi-structured interview during the Q sort)

S4: R report from qmethod package

S5: Summary of bee management practice data

Supplementary Information 1: Pre-pilot interview schedule

Title of Project: Sustainable Beekeeping: Opinion and Practice of Beekeepers in Southwest England

Name of Researcher: Fay Kahane MSc MCIEEM

Stage 1 Pre-pilot Interview Schedule

1. Why do you keep bees?
2. What are your management priorities and goals in beekeeping?
3. Are there any threats to bees or to beekeeping?
4. What does 'sustainable beekeeping' mean to you?

INFORMATION AND CONSENT FORM

Title of Project: Sustainable Beekeeping: Opinion and Practice of Beekeepers in Southwest England (Stage 1)

Name of Researcher: Fay Kahane MSc MCIEEM

I've been a local professional ecologist since 2004 and am currently working on the above project with the Bee Research Group at the University of Exeter in Penryn. If you'd like to take part, this interview will comprise several open-ended questions about why you keep bees and your beekeeping goals and priorities. My aim is to gather information to design a second set of in-depth interviews with Southwest beekeepers (Stage 2). Your data will be fully confidential (including from the rest of the research group) and stored securely on encrypted computers. Contact details will not be released to third parties and will be permanently deleted after 3 years, although you can request deletion at any time by contacting me (as above). Results may be published in an academic journal / beekeeping magazine; data will only be released in a way such that no individual's answers can be identified. It is hoped this study will guide future research priorities and action for local bees and beekeepers.

☐ I confirm that I understand the information above and would like to take part in this interview. I understand that my participation is voluntary and I can withdraw at any time.

☐ I am happy to be contacted again by Fay for participation in Stage 2 interviews for this project. I understand I'm under no obligation to take part in Stage 2.

Name of participant…………………………… Date………………
Signature…………………………..

Name of researcher taking consent………………………………..... Date………………
Signature…………………………..

Supplementary Information 2: Participant Information Sheet and Consent Form

Ethical approval granted 6/6/19 by College of Life & Environmental Sciences Penryn Ethics Committee.

Fay Kahane MSc MCIEEM
Environment & Sustainability Institute
University of Exeter, Penryn Campus, Penryn, Cornwall. TR10 9FE
Tel: 07340 587033
Email: f.kahane@exeter.ac.uk
Website: www.exeter.ac.uk/esi
LinkedIn: www.linkedin.com/in/faykahane

An invitation to take part in a local research project

Title: Why do beekeepers do what they do? Opinion and practice of beekeepers in Southwest England.
Researcher name: Fay Kahane MSc MCIEEM

Introduction: There are many different reasons why people keep bees and many different ways to manage them; if you ask 10 beekeepers you'll get 11 different answers! This small research project seeks to explore the diversity of opinion and practice in the Southwest, and explore why beekeepers do what they do.

Who is organising and funding this study? I've been a local professional ecologist since 2004 and am currently working on the above project with the Bee Research Group at the University of Exeter in Penryn. The project is funded by The Halpin Trust; a grant-making charity that funds projects which deliver a powerful and lasting impact in education, the environment and healthcare: http://www.halpintrust.org.

Purpose of the research: Why and how you manage your bees is very important in a variety of ways; economically, socially and environmentally. It is hoped this study will stimulate further open dialogue within the beekeeping community about the many ways of keeping bees, and explore future research priorities and actions for local bees and beekeepers.

Why have you been approached: This research aims to be in-depth, with a carefully selected sample of beekeepers in Southwest England, chosen to represent a likely variety of viewpoints. I aim to interview around 25-35 beekeepers in total.

What would taking part involve? We'll sit down at a table (I can provide!) and I'll give you around 40 cards with simple statements written on them. Each statement forms the second half of a sentence starting "*As a beekeeper, I do what I do because…*" Example statements include "*I enjoy it*" or "*I like to have calm bees*". I'll ask you to place each statement in a grid like the one below, ordered from most agree to most disagree. I'll encourage you to talk about what you're doing and/or to explain your thoughts after you've completed the grid (everyone likes to do it differently). I'll then ask you around 10 simple, structured questions about your beekeeping (e.g. how many colonies you have, how long you have kept bees). The process is likely to last around an hour but can be longer if you have the time/inclination! It can take place wherever you prefer – your home, place of work, a public café or here at the University campus. I would like to record the interview if you are happy as it makes qualitative analysis more accurate, but I can just take some notes if you prefer. Your answers will be completely confidential of course (more information below), and you can stop at any point. You will receive an electronic copy of the final, anonymised results (if you like) and will be invited to a presentation on the results of the research, likely to be held at the end of the year at the University of Exeter, Penryn.

Example grid (for 22 statements):

Disagree			Neutral			Agree

How will your information be kept confidential? Your answers will be anonymised (including from the rest of the research group) and stored securely on encrypted computers. Contact details and audio files will not be released internally or to third parties and will be permanently deleted after 3 years, although you can request deletion at any time by contacting me (details above). Results may be published in an academic journal / beekeeping magazine; data (including any quotes) will only be released in a way such that no individual's answers can be identified.

Who has reviewed this study? This project has been approved by the College of Life and Environmental Science Research Ethics Committee at the University of Exeter.

Other contacts:
If you wish to give feedback / are not happy with any aspect of the project, please contact Gail Seymour, Research Ethics and Governance Manager g.m.seymour@exeter.ac.uk / 01392 726621.

If you wish for your contact details or audio file to be erased before 3 years, please contact me whilst the project is running (until end 2019), or Juliet Osborne j.l.osborne@exeter.ac.uk from 2020.

If you have any further queries regarding data protection, please ask me or email dataprotection@exeter.ac.uk.

☐ I confirm that I understand the information above and would like to take part in this interview. I understand that my participation is voluntary and I can withdraw at any time.

☐ I am happy for the interview to be recorded. I understand my data will be anonymised; the audio file will be stored securely on encrypted computers and permanently deleted after 3 years (sooner if I request this).

☐ I would like to be invited to a presentation of the results (likely to be held towards the end of 2019 at the Penryn Campus).

Name of participant…………………………………… Date……………… Signature…………………………………………

Thank you very much for your time and interest in this project

Supplementary Information 3: Structured Management & Demographic Questions

Fay Kahane MSc MCIEEM
Environment & Sustainability Institute
University of Exeter, Penryn Campus, Penryn, Cornwall. TR10 9FE
Tel: 07340 587033 Email: f.kahane@exeter.ac.uk
Website: www.exeter.ac.uk/esi LinkedIn: www.linkedin.com/in/faykahane

Why do beekeepers do what they do?
Opinion and practice of beekeepers in Southwest England

1. How would you describe yourself as a beekeeper?

Prompts: *Hobbyist, commercial, farmer, urban*
Sustainable/natural/non-active, active/traditional/conventional, researcher
Novice, amateur, professional, master...

2. Are you a member of any groups or associations?

Prompts: *BBKA, BFA, CBKA, WCBKA, B4, BIBBA, NBKT, local BKAs ...*
How active – how often meetings?

3. How long ago did you first begin keeping bees?

4. How many colonies do you currently own? In how many apiaries?

5. What type of land is/are your apiary(s) in?

Prompts: *Garden (private: urban, suburban, rural), public garden, park, agricultural land, derelict land, scrub, heath, moor, young / mature woodland, coastal...*

6. What type of hive(s) do you use?

And do you use pre-printed foundation?

7. Where do you source your queens / stock?

Prompts: *Imports, raise own - How select? Selecting for what? Artificial insemination?*

8. Do you supplement-feed your bees?

Prompts: *Carbs, protein, sugar, fondant, honey, ambrosia. When? (How often?) Season dependent?*

9. Do you try to manage swarming? How?

Prompts: *Space, ventilation, regular inspection, replace queens, split colony, clip queens, cut out queen cells, split colony / artificial swarm*

10. Do you influence the forage available for your bees? How?

Prompts: *Moving colonies to benefit from particular source? Which?*
On own land: planting flowers, cutting regime
Interactions with landowner(s): planting, pesticides, cutting regime
Interactions with other beekeepers: competition for forage?

11. Do you treat / manage for pests or diseases? How?

Prompts: *Varroa: chemical treatments (which?), natural treatments (which?), hive manipulations (which?) How much / often?*
Diseases: chalkbrood, sacbrood, foulbroods, nosema, viruses (e.g. CBPV)

12. How often do you inspect?

13. Are you registered on BeeBase?

Age: 18-19 20-29 30-39 40-49 50-59 60-69 over 70

Gender:

Any income from beekeeping:

Main income	I make a bit of money	I spend more than I earn
Secondary income	I roughly break even	

Supplementary Information 4: R report from qmethod package

```
Q-method analysis.
Finished on:             Mon Sep 16 14:52:07 2019
Original data:           43 statements, 21 Q-sorts
Forced distribution:     TRUE
Number of factors:       5
Rotation:                varimax
Flagging:                automatic
Correlation coefficient: pearson
```

Original data :

	P1	P2	P3	P4	P5	P6	P7	P8	P9	P10	P11	P12	P13	P14	P15	P16	P17	P18	P19	P20	P21
1	3	2	3	0	2	2	-2	4	4	4	3	-1	3	4	2	1	1	4	2	0	2
2	4	3	4	3	2	2	0	3	3	4	3	1	3	2	0	1	4	4	3	3	2
3	0	0	-1	0	2	-3	-1	0	2	2	1	-2	1	-1	0	2	1	0	0	-2	1
4	0	2	-1	1	0	4	4	-1	0	-2	2	2	0	-1	2	3	1	0	2	-3	0
5	2	-2	0	-1	-3	0	2	0	0	3	1	-3	-2	1	-2	-1	-2	2	-1	-3	0
6	-4	-4	-4	-2	-2	-3	-1	-3	-4	-4	-4	-3	-3	-3	-4	-4	0	-3	-4	-4	-1
7	0	0	0	-4	1	0	3	1	-1	2	3	0	-1	-2	-3	2	3	0	0	-2	0
8	-3	0	4	-1	-1	-2	0	0	-1	0	-1	4	-1	-2	-1	-2	-2	-2	-2	-1	2
9	-1	-1	2	3	4	1	3	1	2	3	4	1	2	1	0	1	0	2	3	1	3
10	-2	-2	-2	-1	-2	-1	-1	0	-2	-2	-1	-2	-1	-4	-1	0	2	-3	4	0	-1
11	-4	-3	2	-2	3	1	-3	-4	-2	-3	-4	2	-1	2	-4	-4	0	-1	-4	-4	4
12	-3	-1	-1	1	-1	1	-1	-2	-1	-2	-1	0	-4	0	-2	-1	-3	-4	-3	1	-3
13	2	1	0	-3	0	1	-1	-3	-3	-4	-1	1	-4	-1	-2	-2	3	-2	-2	-2	3
14	1	-1	-1	-3	2	-1	-4	-3	-1	0	-2	-1	-3	-2	-1	0	2	-2	0	0	-2
15	3	-1	0	-3	-3	-1	-3	-2	-3	-3	-1	-2	-2	1	-2	-2	2	-1	1	-1	2
16	1	1	3	0	-1	0	-2	1	3	2	0	-1	2	3	0	1	0	3	1	1	3
17	-3	-2	3	1	3	0	3	0	1	0	0	1	2	3	-3	-3	0	2	1	-3	4
18	1	-3	-2	-3	-3	-4	-2	-1	0	-3	4	-4	1	-3	-1	-3	4	-2	3	0	-1
19	-3	-3	-3	-2	-2	-3	-2	-4	-3	-2	-2	-3	-1	-4	-1	-2	-4	-4	4	-3	-2
20	-2	1	-1	-1	-1	-1	-2	-1	-2	0	-3	-2	0	-1	1	0	-1	-3	-3	-1	-2
21	0	-2	-2	-4	1	-1	-1	-1	-4	-3	-3	2	2	-1	0	-1	-1	-3	-1	3	0
22	0	0	-3	0	-2	-1	3	-1	0	-2	-1	-2	-2	0	-1	0	-2	-1	-2	3	-1
23	-2	-4	-3	-2	-3	-4	-4	-3	-2	0	-3	-4	-2	-3	-2	-1	-1	-1	-3	-2	-2

24	1	0	2	-2	-1	-2	2	-2	-3	-1	0	3	-1	3	4	-1	-4	-1	-3	-1	-3
25	0	1	0	0	0	-1	1	1	4	1	0	3	3	-1	1	2	-2	0	0	0	1
26	2	-1	1	3	3	3	0	2	1	2	2	2	4	2	1	-1	1	3	1	0	3
27	-2	-3	1	-1	0	-2	0	-2	-1	-1	-2	0	-3	-2	-3	-3	-3	1	-1	-1	1
28	2	1	-2	-1	0	-2	1	2	-1	-1	1	-1	1	2	-1	0	1	1	-2	-2	-1
29	4	1	1	1	4	3	2	1	2	3	3	-1	1	1	1	0	3	1	1	1	1
30	3	4	1	2	3	2	2	2	3	3	1	-1	2	1	2	3	3	2	2	2	1
31	1	3	2	0	1	2	1	4	0	1	1	-1	1	0	1	1	-1	2	1	1	1
32	-1	3	1	3	0	4	0	2	1	1	0	0	-1	0	2	4	1	1	3	2	0
33	-2	3	-4	2	1	-2	2	3	0	1	-2	1	0	-2	0	3	-3	0	0	-1	0
34	-1	-1	-1	2	0	-3	4	1	1	1	1	-3	0	-3	0	-3	-1	-1	-1	0	-1
35	1	0	-2	0	1	1	1	3	-1	0	-2	0	4	-1	3	3	1	1	1	-1	0
36	-1	1	1	2	1	3	0	3	3	2	2	1	0	2	3	4	2	0	2	4	-1
37	1	0	1	4	2	1	1	1	1	1	0	2	3	3	2	0	0	0	-2	2	-4
38	-1	-2	-1	-1	-4	3	-3	-2	2	-1	-1	4	-2	4	1	2	-1	-2	0	1	-4
39	3	4	0	4	-1	0	1	0	1	-1	2	3	1	0	4	1	2	3	2	2	1
40	-1	-1	0	2	-4	0	-1	0	1	-1	1	0	1	1	3	-1	-1	1	0	3	-3
41	-1	2	2	1	-2	1	1	-1	-2	-1	2	1	-3	0	-3	-2	-3	1	-1	1	-2
42	0	2	-3	1	-1	0	-3	-1	0	0	-3	3	0	1	3	2	0	-1	-1	2	-3
43	2	2	3	1	1	2	0	2	2	1	0	0	0	0	1	1	-2	3	-1	4	2

Q-sort factor loadings :

	f1	f2	f3	f4	f5
P1	0.2937	0.217	0.3147	0.653	-0.0578
P2	0.3547	0.667	0.1622	0.170	0.1510
P3	0.3442	-0.052	0.7663	0.045	0.2904
P4	0.6228	0.484	0.1924	-0.149	0.0316
P5	0.3104	0.168	0.1627	0.122	0.7453
P6	0.1248	0.556	0.4906	0.185	0.2667
P7	0.6146	0.131	-0.1668	-0.271	0.3560
P8	0.7406	0.401	0.0750	0.214	0.1644
P9	0.6892	0.324	0.3252	0.223	-0.0014
P10	0.7673	0.189	0.2236	0.170	0.1038
P11	0.6780	0.046	0.1923	0.415	0.0164
P12	-0.1663	0.570	0.4672	-0.316	0.3125
P13	0.5460	0.303	0.1129	0.256	0.2399
P14	0.1733	0.248	0.8226	0.025	0.0062
P15	0.2708	0.786	0.0974	0.121	-0.2185
P16	0.2912	0.798	-0.1103	0.228	0.0371
P17	-0.0052	0.090	0.0085	0.859	0.2479
P18	0.6936	0.106	0.4766	0.236	0.2031
P19	0.3919	0.225	-0.1566	0.587	0.0646
P20	0.2607	0.560	0.2849	0.142	-0.3700
P21	0.2027	-0.270	0.2904	0.263	0.7273

```
Flagged Q-sorts :
    flag_f1 flag_f2 flag_f3 flag_f4 flag_f5
P1  "FALSE" "FALSE" "FALSE" " TRUE" "FALSE"
P2  "FALSE" " TRUE" "FALSE" "FALSE" "FALSE"
P3  "FALSE" "FALSE" " TRUE" "FALSE" "FALSE"
P4  " TRUE" "FALSE" "FALSE" "FALSE" "FALSE"
P5  "FALSE" "FALSE" "FALSE" "FALSE" " TRUE"
P6  "FALSE" "FALSE" "FALSE" "FALSE" "FALSE"
P7  " TRUE" "FALSE" "FALSE" "FALSE" "FALSE"
P8  " TRUE" "FALSE" "FALSE" "FALSE" "FALSE"
P9  " TRUE" "FALSE" "FALSE" "FALSE" "FALSE"
P10 " TRUE" "FALSE" "FALSE" "FALSE" "FALSE"
P11 " TRUE" "FALSE" "FALSE" "FALSE" "FALSE"
P12 "FALSE" "FALSE" "FALSE" "FALSE" "FALSE"
P13 " TRUE" "FALSE" "FALSE" "FALSE" "FALSE"
P14 "FALSE" "FALSE" " TRUE" "FALSE" "FALSE"
P15 "FALSE" " TRUE" "FALSE" "FALSE" "FALSE"
P16 "FALSE" " TRUE" "FALSE" "FALSE" "FALSE"
P17 "FALSE" "FALSE" "FALSE" " TRUE" "FALSE"
P18 " TRUE" "FALSE" "FALSE" "FALSE" "FALSE"
P19 "FALSE" "FALSE" "FALSE" " TRUE" "FALSE"
P20 "FALSE" " TRUE" "FALSE" "FALSE" "FALSE"
P21 "FALSE" "FALSE" "FALSE" "FALSE" " TRUE"

Statement z-scores :
   zsc_f1 zsc_f2 zsc_f3 zsc_f4                  zsc_f5
1    1.69  0.780   1.90  0.894  1.0724259161798295192
2    1.81  0.739   1.51  2.146  1.0724259161798295192
3    0.44  0.249  -0.53  0.346  0.8153583358580549501
4    0.12  0.956  -0.53  0.535  0.0000000000000000043
5    0.53 -0.999   0.31 -0.547 -0.8374361333044206823
6   -1.88 -2.246  -1.82 -0.856 -0.8153583358580549501
7    0.35 -0.303  -0.61  1.038  0.2791453777681401904
8   -0.41 -0.651   0.28 -1.240  0.2349897828754089202
9    1.48  0.162   0.76  0.163  1.8877842520378844693
10  -0.85 -0.399  -1.68  0.830 -0.8153583358580549501
```

11	-1.58	-2.139	1.06	-0.856	1.8657064545915187370
12	-1.06	-0.599	-0.22	-1.680	-1.0503481187334637870
13	-1.61	-0.800	-0.31	1.089	0.7712027409653237076
14	-1.22	-0.291	-0.84	0.812	0.0441555948927312633
15	-1.35	-0.943	0.31	1.146	-0.3233009726608714884
16	0.76	0.378	1.60	0.214	0.4920573631971835726
17	0.56	-1.577	1.60	-0.265	1.8657064545915187370
18	-0.54	-1.098	-1.37	1.787	-1.0945037136261952515
19	-1.58	-1.120	-1.90	-1.367	-1.0724259161798295192
20	-0.88	0.218	-0.53	-0.868	-0.7932805384116893288
21	-1.40	-0.194	-0.76	-0.440	0.2791453777681401904
22	-0.39	0.035	-0.67	-0.881	-0.8153583358580549501
23	-1.19	-1.141	-1.60	-0.868	-1.3515712939479695986
24	-0.66	0.465	1.37	-1.547	-1.0503481187334637870
25	0.71	0.686	-0.31	-0.692	0.2570675803217745692
26	1.24	-0.121	0.84	0.680	1.6086388742697443899
27	-0.65	-1.539	-0.39	-1.372	0.2570675803217745692
28	0.20	-0.222	0.17	0.397	-0.2570675803217745692
29	1.10	0.364	0.53	1.612	1.3736490913943353309
30	1.31	1.536	0.53	1.586	1.0945037136261952515
31	0.83	0.777	0.45	-0.132	0.5362129580899147596
32	0.59	1.625	0.22	0.509	0.0000000000000000043
33	0.49	0.842	-1.51	-1.278	0.2791453777681401904
34	0.63	-0.699	-1.15	-0.560	-0.2570675803217745692
35	0.38	1.070	-0.76	0.560	0.2791453777681401904
36	1.01	1.740	0.84	0.761	0.0220777974463656386
37	0.72	0.513	1.15	-0.069	-0.4699795657508178404
38	-0.71	0.436	1.01	-0.466	-2.1448518323596590385
39	0.69	1.509	0.00	1.240	-0.0220777974463656282
40	0.23	0.465	0.31	-0.466	-1.8877842520378844693
41	-0.15	-0.657	0.45	-1.252	-1.0724259161798295192
42	-0.51	1.307	-0.37	-0.094	-1.0503481187334637870
43	0.75	0.888	0.67	-0.547	0.7932805384116893288

Statement factor scores :

	fsc_f1	fsc_f2	fsc_f3	fsc_f4	fsc_f5
1	4	2	4	2	2
2	4	1	3	4	2
3	0	0	-1	1	2
4	0	2	-1	1	0
5	1	-2	1	-1	-2
6	-4	-4	-4	-2	-1
7	0	-1	-1	2	1
8	-1	-1	0	-2	0
9	3	0	2	0	4
10	-2	-1	-3	2	-1
11	-3	-4	2	-2	3
12	-2	-1	0	-4	-2
13	-4	-2	-1	2	2
14	-2	-1	-2	2	0
15	-3	-2	1	3	-1
16	2	1	3	0	1
17	1	-3	3	0	3
18	-1	-2	-3	4	-3
19	-3	-3	-4	-3	-3
20	-2	0	-1	-2	-1
21	-3	0	-2	0	1
22	-1	0	-2	-2	-1
23	-2	-3	-3	-2	-3
24	-1	1	3	-4	-2
25	1	1	-1	-1	0
26	3	0	2	1	3
27	-1	-3	-1	-3	0
28	0	-1	0	1	-1
29	3	0	1	3	3
30	3	3	1	3	3
31	2	2	1	0	1
32	1	4	0	1	0
33	1	2	-3	-3	1
34	1	-2	-2	-1	-1
35	0	3	-2	1	1
36	2	4	2	1	0

37	2	1	3	0	-1
38	-1	1	2	-1	-4
39	1	3	0	3	0
40	0	1	1	-1	-4
41	0	-1	1	-3	-3
42	-1	3	-1	0	-2
43	2	2	1	-1	2

Factor characteristics:

General factor characteristics:

	av_rel_coef	nload	eigenvals	expl_var	reliability	se_fscores
f1	0.8	8	4.5	22	0.97	0.17
f2	0.8	4	3.6	17	0.94	0.24
f3	0.8	2	2.6	12	0.89	0.33
f4	0.8	3	2.4	11	0.92	0.28
f5	0.8	2	1.9	9	0.89	0.33

Correlation between factor z-scores:

	zsc_f1	zsc_f2	zsc_f3	zsc_f4	zsc_f5
zsc_f1	1.00	0.617	0.54	0.41	0.479
zsc_f2	0.62	1.000	0.28	0.34	0.062
zsc_f3	0.54	0.284	1.00	0.18	0.412
zsc_f4	0.41	0.345	0.18	1.00	0.367
zsc_f5	0.48	0.062	0.41	0.37	1.000

Standard error of differences between factors:

	f1	f2	f3	f4	f5
f1	0.25	0.30	0.38	0.33	0.38
f2	0.30	0.34	0.41	0.37	0.41
f3	0.38	0.41	0.47	0.43	0.47
f4	0.33	0.37	0.43	0.39	0.43
f5	0.38	0.41	0.47	0.43	0.47

Distinguishing and consensus statements:

Statement no.	Distinguishing (D) / Consensus	f1_f2	sig_f1_f2	f1_f3	sig_f1_f3	f1_f4	sig_f1_f4	f1_f5	sig_f1_f5	f2_f3	sig_f2_f3	f2_f4	sig_f2_f4	f2_f5	sig_f2_f5	f3_f4	sig_f3_f4	f3_f5	sig_f3_f5	f4_f5	sig_f4_f5
1		0.91	**	-0.21		0.80	*	0.62		-1.12	**	-0.11		-0.29		1.01	*	0.83		-0.18	
2		1.07	***	0.30		-0.33		0.74	*	-0.77		-1.41	***	-0.33		-0.63		0.44		1.07	*
3		0.19		0.97	*	0.09		-0.38		0.78		-0.10		-0.57		-0.88	*	-1.35	**	-0.47	
4		-0.83	**	0.66		-0.41		0.12		1.49	***	0.42		0.96	*	-1.07	*	-0.53		0.54	
5		1.52	****	0.22		1.07	**	1.36	***	-1.31	**	-0.45		-0.16		0.85	*	1.15	*	0.29	
6		0.36		-0.06		-1.03	**	-1.07	**	-0.43		-1.39	***	-1.43	***	-0.96	*	-1.00	*	-0.04	
7		0.65	*	0.96	*	-0.69	*	0.07		0.31		-1.34	***	-0.58		-1.65	***	-0.89		0.76	
8		0.24		-0.70		0.83	*	-0.65		-0.93	*	0.59		-0.89	*	1.52	***	0.05		-1.48	***
9		1.32	***	0.72		1.32	***	-0.41		-0.59		0.00		-1.73	***	0.59		-1.13	*	-1.73	***
10	D f4	-0.45		0.83	*	-1.68	****	-0.03		1.28	**	-1.23	***	0.42		-2.51	****	-0.86		1.65	***
11	D f4	0.56		-2.65	****	-0.73	*	-3.45	****	-3.20	****	-1.28	***	-4.00	****	1.92	***	-0.80		-2.72	****
12		-0.46		-0.84	*	0.62		-0.01		-0.38		1.08	**	0.45		1.46	***	0.83		-0.63	
13	D f1	-0.81	**	-1.31	***	-2.70	****	-2.38	****	-0.49		-1.89	****	-1.57	***	-1.40	**	-1.08	*	0.32	
14		-0.93	**	-0.38		-2.03	****	-1.27	***	0.55		-1.10	**	-0.34		-1.65	***	-0.88		0.77	
15		-0.41		-1.66	***	-2.50	****	-1.03	**	-1.25	**	-2.09	****	-0.62		-0.84		0.63		1.47	***
16	D f3 only	0.38		-0.84	*	0.55		0.27		-1.22	**	0.16		-0.11		1.38	**	1.10	*	-0.28	
17	D f1, f2, f4	2.14	****	-1.04	**	0.83	*	-1.31	***	-3.17	****	-1.31	***	-3.44	****	1.86	***	-0.27		-2.13	****
18	D f4	0.56		0.83	*	-2.33	****	0.56		0.27		-2.89	****	0.00		-3.16	****	-0.28		2.88	****
19	Consensus	-0.46		0.32		-0.22		-0.51		0.78		0.25		-0.05		-0.54		-0.83		-0.29	
20		-1.10	***	-0.35		-0.02		-0.09		0.75		1.09	**	1.01	*	0.34		0.26		-0.08	
21		-1.20	***	-0.64		-0.96	**	-1.68	***	0.56		0.25		-0.47		-0.32		-1.04	*	-0.72	
22		-0.42		0.29		0.50		0.43		0.71		0.92	*	0.85	*	0.21		0.14		-0.07	
23	Consensus	-0.05		0.41		-0.32		0.16		0.45		-0.77		0.21		-0.73		-0.74		0.48	
24	D f2, f3	-1.13	***	-2.03	****	0.89	**	0.39		-0.91	*	2.01	****	1.52	***	2.92	****	2.42	****	-0.50	
25		0.03		1.02	**	1.40	***	0.45		0.99	*	1.38	***	0.43		0.39		-0.57		-0.95	*
26	D f2	1.36	***	0.40		0.56		-0.37		-0.96	*	-0.80		1.73	***	0.16		-0.77		-0.93	*

Statement no.	Distinguishing (D) / Consensus	f1_f2	sig_f1_f2	f1_f3	sig_f1_f3	f1_f4	sig_f1_f4	f1_f5	sig_f1_f5	f2_f3	sig_f2_f3	f2_f4	sig_f2_f4	f2_f5	sig_f2_f5	f3_f4	sig_f3_f4	f3_f5	sig_f3_f5	f4_f5	sig_f4_f5
27		0.89	**	-0.26		0.73	*	-0.90	*	-1.15	**	-0.17		-1.80	***	0.98	*	-0.65		-1.63	***
28	Consensus	0.42		0.04		-0.20		0.46		-0.39		-0.62		0.04		-0.23		0.42		0.65	
29		0.74	*	0.57		-0.51		-0.27		-0.17		-1.25	***	-1.01	*	-1.08	*	-0.84		0.24	
30		-0.22		0.78	*	-0.27		0.22		1.00	*	-0.05		0.44		-1.05	*	-0.56		0.49	
31		0.05		0.38		0.96	**	0.29		0.33		0.91	*	0.24		0.58		-0.09		-0.67	
32	D f2 only	-1.03	***	0.37		0.08		0.59		1.40	***	1.12	**	1.63	***	-0.29		0.22		0.51	
33		-0.35		2.00	****	1.77	****	0.21		2.35	****	2.12	****	0.56		-0.23		-1.79	***	-1.56	***
34	D f1 only	1.33	***	1.78	***	1.19	***	0.89	*	0.45		-0.14		-0.44		-0.59		-0.89		-0.30	
35	D f3	-0.69	*	1.14	**	-0.18		0.11		1.83	***	0.51		0.79		-1.32	**	-1.04	*	0.28	
36	D f2	-0.73	*	0.17		0.25		0.99	**	0.90	*	0.98	**	1.72	***	0.08		0.82		0.74	
37		0.21		-0.43		0.79	*	1.19	**	-0.63		0.58		0.98	*	1.22	**	1.62	***	0.40	
38	D f5	-1.14	***	-1.71	***	-0.24		1.44	***	-0.57		0.90	*	2.58	****	1.47	***	3.15	****	1.68	***
39		-0.82	**	0.69		-0.55		0.72		1.51	***	0.27		1.53	***	-1.24	**	0.02		1.26	**
40	D f5	-0.24		-0.08		0.69	*	2.12	****	0.16		0.93	*	2.35	****	0.77		2.20	***	1.42	**
41		0.50		-0.60		1.10	***	0.92	*	-1.11	**	0.59		0.42		1.70	***	1.52	**	-0.18	
42	D f2	-1.82	****	-0.15		-0.42		0.54		1.67	***	1.40	***	2.36	****	-0.27		0.69		0.96	*
43	D f4 only	-0.14		0.08		1.30	***	-0.04		0.22		1.43	***	0.10		1.22	**	-0.12		-1.34	**

Supplementary Information 5. Summary of bee management practice data.

* Denotes a self-described natural beekeeper. Number in parentheses indicates number of participants that practice a management form.

	Frequency of internal inspection	Chemical *Varroa* treatment	Swarm management	Source of stock	Drone culling
**Conventional hobbyists n=8**	Every 7-10 days (7)<br>Around once a month (1)*	Prophylactic (7)<br>None (1)*	Yes (7)<br>No (1)*	Local / raise own queens (7)<br>UK supplier (1)	Yes (2)<br>Reducing (2)<br>No (1)
**Natural beekeepers n=4**	Around once a month (4)	None (4)	No (4)	Local (3)<br>Local supplier (1)	No (4)
**Black bee farmers n=4**	Every 7-10 days (3)<br>Every 14 days (1)	As needed: all colonies (2)<br>Prophylactic: productive colonies / as needed: breeder colonies (2)	Yes (4)	Raise own queens (4)	No (3)<br>Reducing (1)
**New-conventional hobbyists n=3**	Every 7-10 days (3)	As needed (2)<br>Prophylactic (1)	Yes (2)<br>No (1)	Local (2)<br>UK source (1)	Reducing (2)<br>No (1)
**Pragmatic bee farmers n=2**	Every 7-10 days (2)	Prophylactic (2)	Yes (2)	Local / raise own queens (1)<br>UK supplier (1)	Reducing (1)<br>No (1)
